# Supplementary figures and images for: Inhibition of SARS-CoV-2 infection in human iPSC-derived cardiomyocytes by targeting the Sigma-1 receptor disrupts cytoarchitecture and beating
Source: PeerJ. 2021 Dec 20;9:e12595. doi: 10.7717/peerj.12595 (PMC8697769; doi:10.7717/peerj.12595)

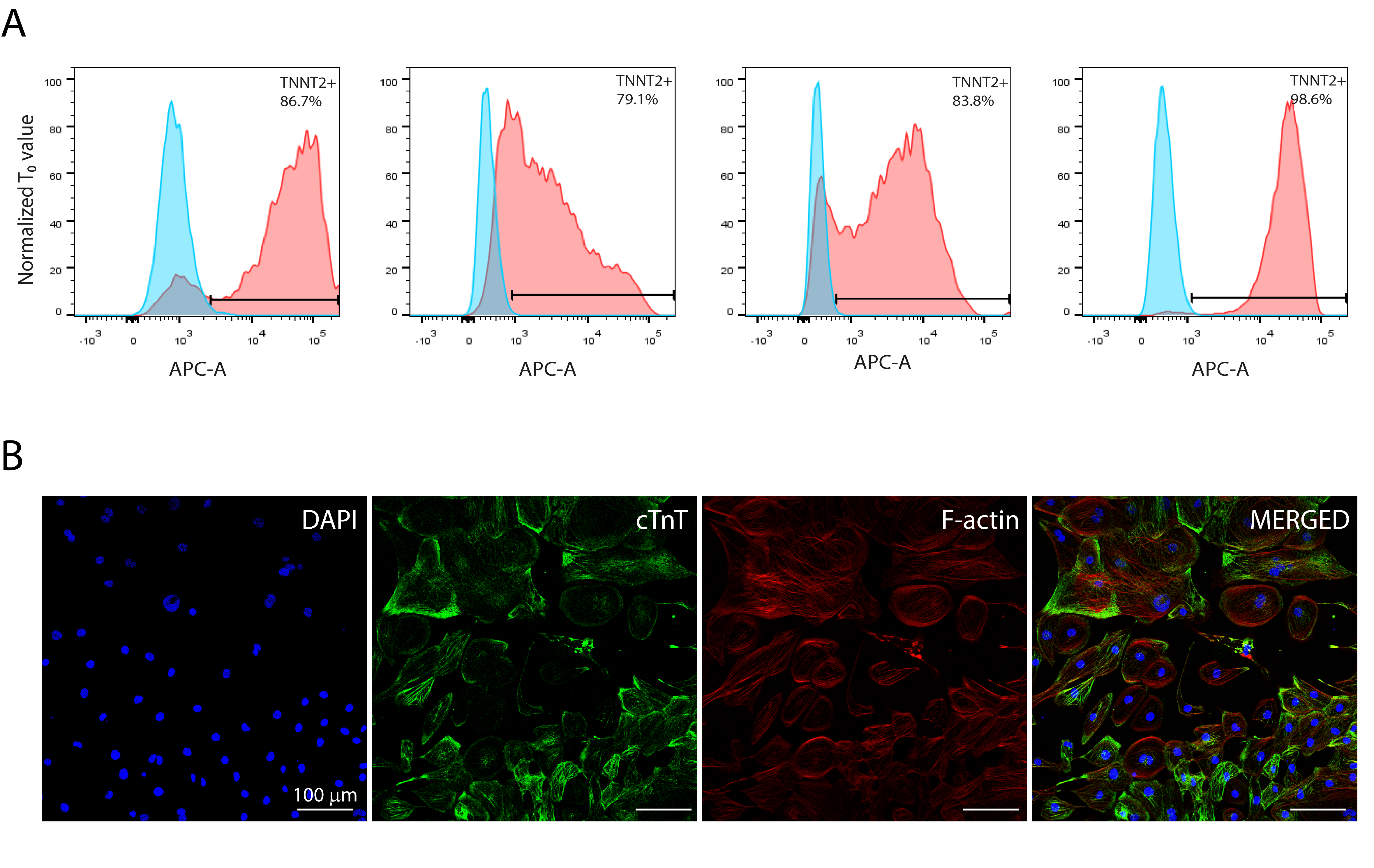

Supplement: Supplemental Information 1 — (A) Flow cytometry data representing the expression of cardiac-specific marker troponin T (cTnT/TNNT2) from four different batches (N = 4), confirming highly efficient differentiation into cardiomyocytes. (B) Immunocytochemistry for cTnT and phalloidin staining for filamentous actin (F-actin). Anti-cTnT (green); phalloidin (red) and nuclei (blue); scale bar = 100 µm. (N = 4; further analyzed in Fig. 4). [file peerj-09-12595-s001.png]

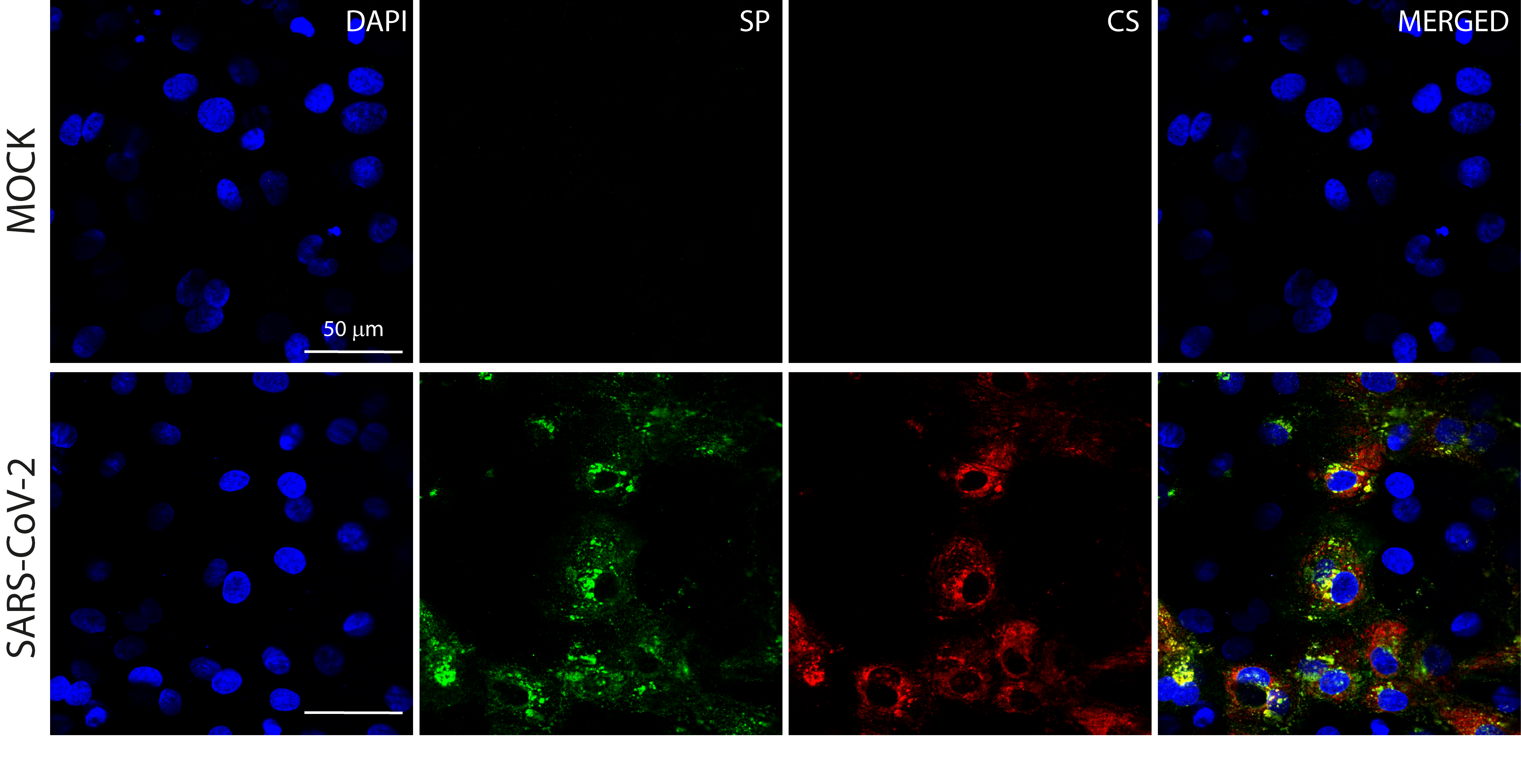

Supplement: Supplemental Information 2 — The CS showed robust and specific immunoreactive signals overlapped with the SP staining in SARS-CoV-2 infected cardiomyocyte cultures (MOI 0.1 at 48 h.p.i), while no such signal was detected in mock-infected condition. The staining was performed in independent experiments (N = 3). Scale bar = 50 µm. [file peerj-09-12595-s002.png]

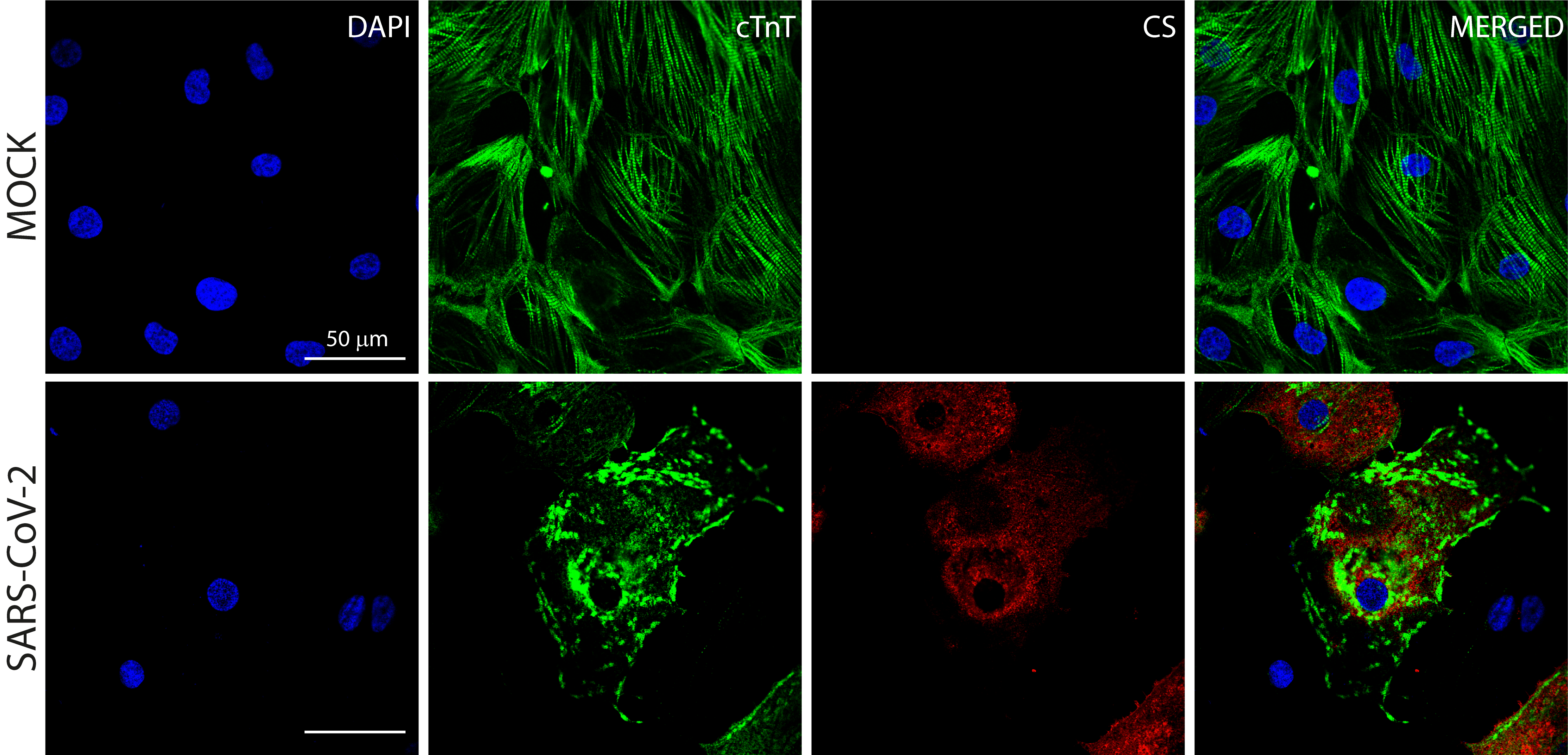

Supplement: Supplemental Information 3 — Representative immunostaining images of cytoskeleton fragmentation of cardiomyocytes exposed to SARS-CoV-2 (MOI 0.1) for 48 h from 4 independent experiments (N = 4). Anti-convalescent serum (CS) positive staining indicates the presence of SARS-CoV-2 in cells exhibiting Cardiac TnT discontinuity and disruption. cTnT (green), CS (red) and nuclei (blue). Scale bar = 50 µm. [file peerj-09-12595-s003.png]

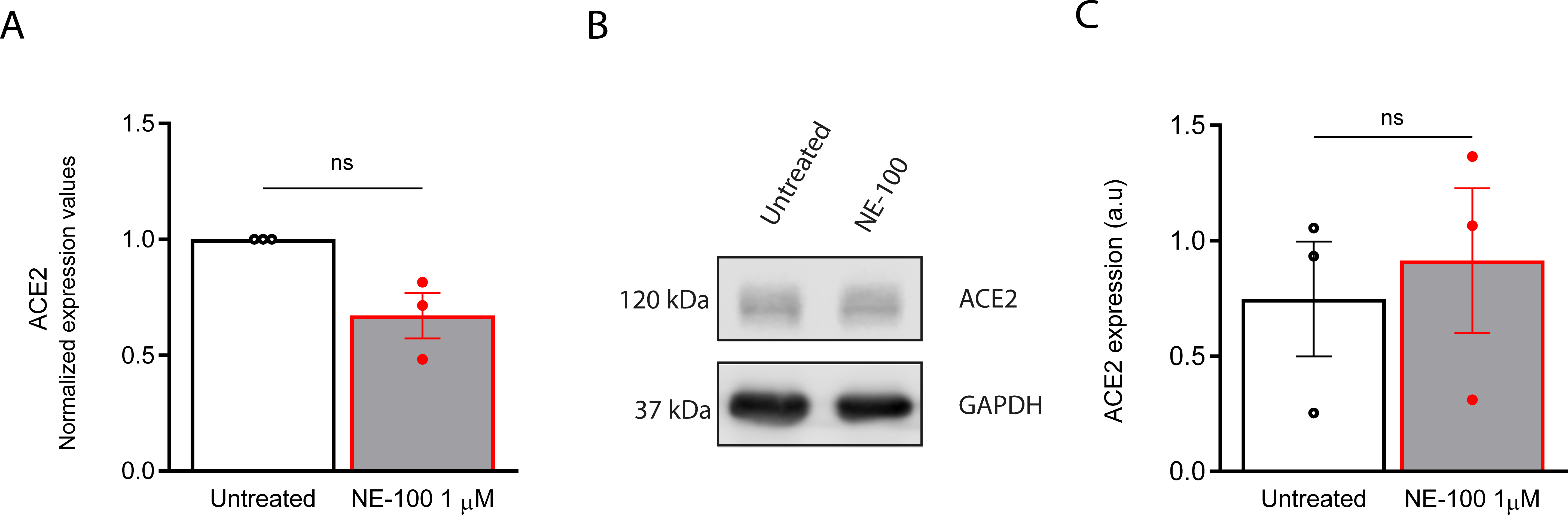

Supplement: Supplemental Information 4 — (A) Real-time PCR shows ACE2 mRNA levels. Expression values are normalized by endogenous control genes GAPDH and HPRT1 and are expressed as fold change relative to control (untreated) condition (N = 3). Data are represented as the mean ± S.E.M relative to the untreated condition analyzed by unpaired Welch’s t test (p = 0.0790). (B) Representative blots for ACE2 in protein extracts from untreated hiPSC-CMs or stimulated with 1 µM of NE-100 for 24 h (N = 3). Full-length gels are shown in Fig. S5. (C) Blots performed in samples from three independent batches were quantified by densitometry and normalized by GAPDH expression (N = 3). Data are represented as the mean ± S.E.M relative to the untreated condition analyzed by unpaired Welch’s t test (p = 0.7006). Data points represent independent experiments. [file peerj-09-12595-s004.png]

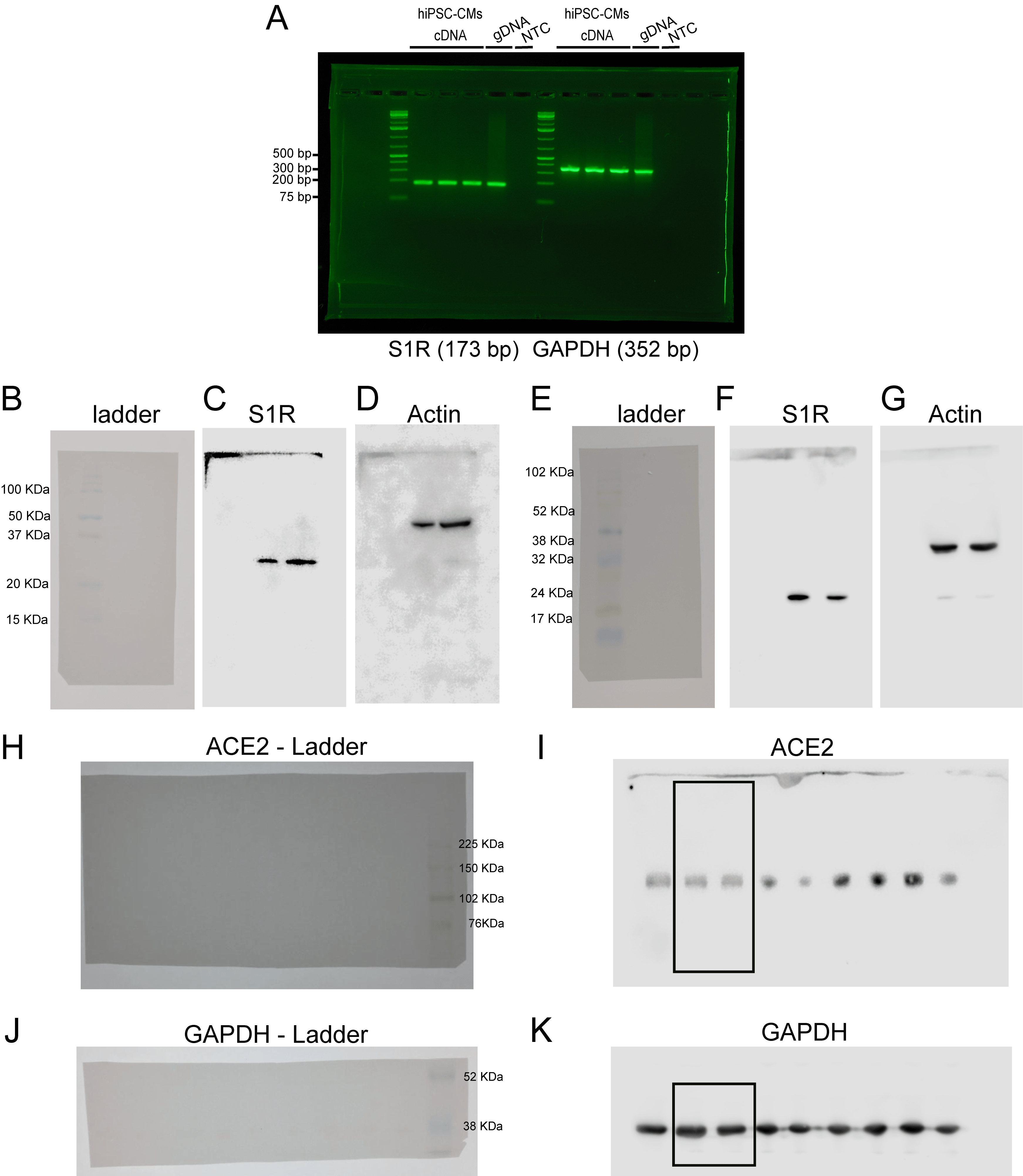

Supplement: Supplemental Information 5 — (A) Agarose gel electrophoresis of PCR products amplified from mRNA/cDNA samples obtained from hiPSC-CMs derived from three distinct batches of differentiation (N = 3). Appropriate genomic DNA (gDNA) positive controls and negative no-template controls (NTC) were incorporated into each reaction. The primer pairs used for S1R and GAPDH detection are presented in the methods section. (B-G) Western blot for detection of Sigma-1 receptor (S1R) in human induced pluripotent stem cell-derived cardiomyocytes (hiPSC-CMs) (N = 3). (B and E)Image of the full-length membrane used for the detection showing the ladder (Amersham ECL Rainbow Marker - Full range) in the first (B) and second (E) experiments. (C and F) Full-length gel of S1R where the two lanes contain replicates of the detection in samples from two different batches (C) and biological replicates of the detection in one batch (F) (D and G) After S1R detection, membranes were submitted to a stripping protocol as described in the methods section and probed for actin. Cropped versions of the membranes (F) and (G) are represented in the Main Fig. 1 Panel B as representative images of S1R detection. (H–K) Western blot for detection of ACE2 in untreated and NE-100 treated hiPSC-CMs. After transference, the membrane was cut, the upper half was used for ACE2 detection, and the bottom half was used for GAPDH detection. (H, J) Images of the membranes used for detection showing the ladder (Amersham ECL Rainbow Marker - Full range). (I,K) Full-length gels for ACE2 and GAPDH. The lanes used for the representative image in Fig. S4 are highlighted in a box. Other lanes are not related to the experiment described here. The experiment was performed three times, using samples from three different batches (N = 3). [file peerj-09-12595-s005.png]
